# Changes in the Lymphoreticular Tissues of Mice Bearing the Landschütz Tumour

**DOI:** 10.1038/bjc.1965.41

**Published:** 1965-06

**Authors:** A. M. El Hassan, A. E. Stuart

## Abstract

**Images:**


### CHANGES IN THE LYMPHORETICULAR TISSUES OF MICE BEARING THE LANDSCHÜTZ TUMOUR

A. M. EL HASSAN AND A. E. STUART

*From The Department of Pathology, University of Edinburgh*

Received for publication November 5, 1964

It is well known that homotransplantable tumours elicit a host response. The purpose of this paper is to study this response over a period of time and to show the early phase of reactivity is followed by inactivity. This later stage can be correlated with atrophy of the thymus and a reduction of spleen weight. Secondly the phagocytic activity of the reticulo-endothelial system (R.E.S.) has been examined by the carbon clearance method with particular reference to the effect of adoptive immunity on R.E.S. function. Lastly the haematological changes in the blood of tumour-bearing animals has been recorded.

### MATERIAL AND METHODS

### *Mice*

100 A/Jax (Porton) and 200 A/Cum (Cumberland Farms) sublines of the inbred strain A mouse weighing 16–18 g. were used.

### *Tumour*

Landschütz ascites tumour was maintained in an outbred but closed colony of mice for 4 years. The tumour was first introduced into A/Jax mice 6 months ago from the outbred mice. Since then it has been maintained within that strain by weekly transfer of fresh ascites fluid in a volume of 0·1–0·5 ml. The A/Cum mice were always inoculated from tumour maintained in A/Jax mice.

### *Changes in the Organs of Mice Bearing Tumour*

Sixty A/Jax mice were inoculated intraperitoneally with a dose of $10^5$ ascites cells. The animals were weighed every 2 days and inspected daily for ascites. Ascites was indicated by an increase in weight of 3 g. over a period of 2 days or by visible abdominal distension.

Groups of 8 mice were killed at 5, 10, 20 and 25 days after tumour inoculation. The animals were first bled from the neck vessels and the blood was used for haematological investigations as described below. A detailed autopsy was then performed. The spleens, livers, thymuses and adrenals were weighed and fixed in neutral formol saline for histological examination. Splenic dabs were made and stained by the Unna Pappenheim method. Some animals bearing the tumour were allowed to die naturally and the organs were inspected and weighed at death. The carcass weight was compared with the initial weight.

*Effect of the site of tumour growth on the host response*

Seven mice were inoculated subcutaneously in the right flank with $10^7$ ascites cells in 0·1 ml. volume. The fluid was cultured aerobically and anaerobically on blood agar plates. Six days later the animals were killed ; the regional lymph nodes, spleens and local tumour were removed and fixed in neutral formol saline for histological examination.

*Estimation of the phagocytic function of the reticulo-endothelial system in mice bearing the Landschütz tumour*

Following the inoculation of $10^5$ ascites cells intraperitoneally (I.P.) in 25 female A/Cum mice the phagocytic function of the reticulo-endothelial system was determined by the carbon clearance method (Biozzi, Stiffel, Halpern, Mouton, 1958). Colloidal carbon was injected intravenously in a dose of 8 mg./100 g. of body weight. Samples of 0·025 ml. of blood were removed from the retro-orbital venous plexus at 3-minute intervals over a period of 15 minutes. The phagocytic coefficient $K$ was calculated from the formula

$$K = \frac{\text{Log } C_1 - \log C_2}{T_2 - T_1}$$

where $C_1$ and $C_2$ are the concentrations of the carbon in the blood at their respective times $T_1$ and $T_2$. Time is expressed in minutes. The corrected phagocytic coefficient $\alpha$ was calculated from the formula

$$\alpha = \sqrt[3]{K} \times \frac{W}{WLS}$$

where $W$ = body weight and $WLS$ is the combined weight of the liver and spleen. An appropriate correction was made for the presence of ascites.

*Phagocytic Function in A/Cum Mice Bearing Tumour but Treated with Immune Isologous Spleen Cells*

The phagocytic function was determined in the following groups of mice.

(1) Fifteen mice bearing $10^5$ ascites cells but treated with $500 \times 10^6$ isologous immune spleen cells 48 hours later. The spleen cells were derived from mice that had been inoculated with $10^5$ cells I.P. 10 days before.

(2) Thirteen mice inoculated with $500 \times 10^6$ immune spleen cells only This group will be called treated controls.

(3) The phagocytic function in the above two groups was compared with that of normal A/Cum mice. This group will be referred to as normal controls.

The spleens, liver, thymuses and adrenals from animals used in the carbon clearance experiments were weighed and examined histologically.

*Haematological Changes in Mice Bearing Tumour*

A/Jax mice were inoculated with $10^5$ ascites cells I.P., bled and killed at 5, 10, 15, 20 and 25 days as described above. The blood was added to 0·025 ml. of heparin and the haemoglobin, total red and white cell count, packed cell volume and differential count were determined. The haemoglobin was determined by the oxy-haemoglobin method using an E.E.L. colorimeter with a green filter. The PCV was determined in micro haematocrit tubes centrifuged at 4500 r.p.m. for 45 minutes. The Coombs antiglobulin test was done as follows. Rabbit anti-mouse serum was absorbed with an equal volume of packed normal mouse red cells which had been washed three times in physiological saline. Absorption was carried out at 37° C. for 60 minutes. Red cells from animals bearing tumour were washed three times in normal saline and made into a 10% suspension in physiological saline. Equal volumes of test red cells and antiglobulin serum were mixed in precipitin tubes and incubated at 37° C. for 30 minutes. Then they were examined microscopically for agglutination.

Marrow smears were made from the right femur and stained with Leishman stain.

*Bacteriological Examination*

Four A/Jax mice were inoculated with $10^5$ ascites cells I.P. Ten days later the animals were killed and under sterile conditions the peritoneal cavity was exposed. The ascites fluid from each mouse was plated directly on two nutrient agar and two blood agar plates. One agar and one blood agar plate was incubated aerobically at 37° C. ; the other two were incubated anaerobically at 37° C.

Aerobic and anaerobic cultures were similarly prepared from the cut surface of the spleens of the above mice. The spleens of 4 normal control mice were also cultured. In addition a Seitz filtrate of ascitic fluid was prepared and 0·1 ml. injected I.P. into 6 mice. They were killed 10 days later and the spleens were weighed.

### RESULTS

*Changes in Organ Weights of Mice bearing Intra-Peritoneal Tumour*

The spleen showed an increase in weight in the early stages of tumour growth. Fig. 1 shows that in A/Jax mice the spleen reached a maximum size 10 days after

FIG. 1.—Average weight of the spleen in groups of 8 mice after intraperitoneal injection of $10^5$ cells. The dotted lines show the limits of standard deviation of 19 controls.

tumour inoculation. It then became smaller until at 25 days it was nearly normal. A similar change in spleen weight was observed with A/Cum mice (Table I). Spleens from mice bearing a 10-day growth of tumour contained 260 $\times$ $10^6$ cells per spleen. Control spleens yielded 160 $\times$ $10^6$ cells per spleen.

TABLE I

| Number of mice | Time after tumour (days) | Average K | Average $a$ | Average spleen wt. (g.) | Average liver wt. (g.) | Average thymus wt. (g.) | Wt. of thymus of control of same age |
|---|---|---|---|---|---|---|---|
| 6 | 5 | ·035 | 4·7 | 0·13 | 1·17 | 0·05 | 0·05 |
| 10 | 10 | ·049 | 5·0 | 0·18 | 1·20 | 0·028 | 0·029 |
| 6 | 15 | ·034 | 4·3 | 0·12 | 1·18 | 0·012 | 0·045 |
| 4 | 21 | ·021 | 3·3 | 0·10 | 1·13 | 0·003 | 0·028 |
| 15 | — | ·032 | 4·5 | 0·10 | 1·07 | 0·039 | — |
| Normal controls | | ±0·011 | ±0·28 | ±0·01 | ±0·08 | ±0·008 | |

Phagocytic indices K and $a$ with organ weights from mice given $10^5$ ascites cells (A/Cum. mice).

In a combined series of 42 A/Jax and A/Cum mice the liver weights were not altered significantly.

The thymus decreased in weight as the tumour grew and in the late stages showed complete atrophy (Fig. 2 and Table I). This was found in both A/Cum and A/Jax mice but especially in the latter. The greatest loss in thymic weight occurred between 15 and 20 days after inoculation of tumour. The variation of A/Cum thymus weights was probably related to age differences.

There was no significant change in adrenal weight of mice bearing the tumour (Table II).

FIG. 2.—Average weight of the thymus in groups of 5 A/Jax mice after intraperitoneal injection of $10^5$ ascites cells. The dotted lines show the limits of standard deviation of 10 controls.

TABLE II.—*Adrenal weight in A/Jax Mice Bearing Tumour*

| Number of mice | | Time after $10^5$ ascites cells (days) | | Adrenal weight | | Average adrenal weight of 10 normal controls |
|---|---|---|---|---|---|---|
| 13 | . | 10 | . | 5·5±1·3 | . | 3·9±0·9 |
| 5 | . | 20 | . | 4·4 | | |

*Morphological Changes in the Lymphoreticular Tissues of Mice Bearing the Tumour*

The spleen showed no obvious changes 5 days after the intra-peritoneal inoculation of tumour. At 10 days the Malpighian bodies were larger than normal. Fig. 3 shows that the normal Malpighian body contained small cells with a round darkly staining nucleus ; the cytoplasm was scanty. Fig. 4 shows that the Malpighian body of spleens from mice bearing a 10-day growth of the tumour was made up of larger cells. The nucleus was larger, irregular, vesicular and contained a single prominent nucleolus. The chromatin was dispersed in irregular clumps or was condensed in a thin rim on the nuclear membrane. Mitotic activity was increased. The cytoplasm was moderate in amount and agranular. The red pulp at 10 days contained foci of proliferating cells with an eccentric or central vesicular nucleus and plentiful pyronophilic cytoplasm. Small spherules of pyronophilic material 1–3 μ in diameter were seen in the sinusoids of the spleen. These globules were homogeneous and opaque. They were entirely separate from the erythrocytes and nucleated cells. Mature plasma cells were not seen. Fifteen days after tumour inoculation the Malpighian bodies were still active ; the red pulp now contained an increased number of granular leucocytes.

In the late stages of tumour growth (20 and 25 days) the splenic activity was markedly decreased. The Malpighian bodies were small and the red pulp showed a marked degree of venous congestion. Many of the cells in the sinusoids were granular leucocytes.

Five days after tumour inoculation the livers of most animals were normal although in some cases the Kupffer cells were stimulated. The Kupffer cell changes were best seen after the intravenous injection of colloidal carbon. At 10 days the Kupffer cells were larger and the cytoplasm more abundant and branched than usual (Fig. 6). The normal pattern is shown in Fig. 5. At this time immature mononuclear cells appeared. They were seen as small clusters of 8–10 cells in the sinusoids or sometimes as individual cells. Larger foci were situated in the portal tracts in intimate relation to a bile duct (Fig. 7) or vein. When situated in the portal tract these foci were well developed and measured up to 100 μ in diameter. Very rarely, these foci developed near a central vein. These mononuclear cells showed a variable morphology and measured between 7 and 10 μ the nuclei were round or slightly indented ; sometimes they were vesicular with a single prominent nucleolus ; in other cells the chromatin was dispersed in minute masses and the cytoplasm was scanty. When the foci were situated in portal tracts, fibroblasts, macrophages and occasional lymphocytes or polymorphs were also seen. In the case of mononuclear cell foci in the vicinity of a vein, the adjacent vascular endothelium was sometimes swollen (Fig. 8).

Mature plasma cells in the liver (Fig. 9) were seen in only one mouse which had multiple tumour deposits in that organ. Scattered in the sinusoids an occasional megakaryocytic type of cell was seen. These cells measured about 20 μ in diameter and had lobulated vesicular nuclei with abundant cytoplasm. In some animals there was an increase in mitotic activity in the hepatic parenchyma cells at 10 days (Fig. 10). At 15 days the Kupffer cells were still active although the foci of mononuclear cells were less prominent. At 20 and 25 days after tumour inoculation the Kupffer cells appeared normal, foci of mononuclear cells were no longer seen, and the sinusoids and blood vessels now contained polymorphonuclear leucocytes.

### *Changes in the Lymphoid Tissue in Response to Subcutaneous Tumour Growth*

The tumour grew as a solid mass showing extensive ischaemic necrosis and a peripheral chronic inflammatory reaction. Moderate numbers of plasma cells were seen as well as lymphocytes, macrophages and mast cells. The regional lymph nodes were enlarged and showed a sinus cell hyperplasia. The marginal sinus and the sinusoids in the depth of the gland were lined by cords of histiocytic cells one to three layers in thickness (Fig. 11). The follicles were large and showed prominent germinal centres and increased mitotic activity (Fig. 12). At this time plasma cells were not seen in the lymph nodes. The spleen and liver from mice bearing subcutaneous tumour were normal.

### *Phagocytic Function in Mice Bearing Tumour*

Fig. 13 shows that when the tumour was inoculated in the peritoneal cavity of A/Cum mice there was a rise of K in the early stages of tumour development and that in the late stages of tumour growth K returned to normal levels. The corrected phagocytic index $\alpha$ was elevated 10 days after tumour inoculation (Table I) When the tumour growth was suppressed by passive immunisation there was no variation in phagocytic function (Fig. 14). Treated control mice showed no change in K or $\alpha$.

### *Haematological Changes in Animals Bearing Tumour*

There was a slight terminal fall in haemoglobin concentration and a polymorphonuclear leukocytosis which was maximal 15 days after tumour inoculation and still present at 25 days. Monocytes were slightly raised in number (Table III). The Coombs test was negative.

---

EXPLANATION OF PLATES

FIG. 3.—Normal Malpighian body of A/Jax spleen. H. & E. ×800.

FIG. 4.—Malpighian body of A/Jax spleen, 10 days after intraperitoneal injection of $10^5$ ascites cells. H. & E. ×800.

FIG. 5.—Kupffer cells of normal animals after intravenous carbon. H. & E. ×340.

FIG. 6.—Kupffer cells of animals bearing $10^5$ ascites cells for 10 days and injected with carbon intravenously.

FIG. 7.—Focus of immature mononuclear cells surrounding a bile duct. H. & E. ×635.

FIG. 8.—Focus of immature mononuclear cells near a portal vein. H. & E. ×400.

FIG. 9.—Mature plasma cells in the portal tract of a mouse bearing tumour. H. & E. ×540.

FIG. 10.—Mitosis in liver cell of a mouse bearing a 10 day growth of $10^5$ ascites cells by the intraperitoneal route. H. & E. ×800.

FIG. 11.—Sinus cell hyperplasia in a lymph node draining a subcutaneous tumour. H. & E. ×240.

FIG. 12.—Prominent germinal centre in a follicle of a lymph node draining a subcutaneous tumour. H. & E. ×160.

3

4

5

6

Hassan and Stuart.

7

8

9

Hassan and Stuart.

10

11

12

FIG. 13.—Phagocytic coefficient $K$ in A/Cum mice after I.P. inoculation of $10^5$ ascites cells. The dotted lines show the limits of standard deviation of 15 normal controls. Each solid circle represents one mouse.

FIG. 14.—Phagocytic coefficient $K$ in A/Cum mice inoculated with tumour and treated with spleen cells. The limits of standard deviation of $K$ for 15 normal controls are shown between dotted lines. Each solid circle represents a single mouse.

TABLE III.—*Changes in peripheral blood leucocytes following inoculation of* $10^5$ *Ascites Cells I.P. in A/Jax Mice*

| Number of mice | Days after tumour inoculation | Total W.B.C./c. mm. Average | Polymorphs | Lymphocytes | Monocytes |
|---|---|---|---|---|---|
| 5 | 5 | 3,552 | 761 | 2,585 | 206 |
| 4 | 10 | 5,428 | 2,567 | 2,604 | 257 |
| 4 | 15 | 12,626 | 9,965 | 2,116 | 545 |
| 5 | 20 | 10.454 | 7,057 | 2,630 | 767 |
| 4 | 25 | 11,043 | 7,600 | 3,270 | 173 |
| Normal values for 10 control mice | | 3,709 | 740 | 2,709 | 260 |

### *Bacteriological Examination*

No organisms were grown from the aerobic or anaerobic cultures of the ascitic fluid or spleens. The Seitz filtrate of ascites fluid did not produce an increase in spleen weight.

### DISCUSSION

Morphological changes in the reticulo-endothelial system of animals bearing transplantable tumours have attracted the attention of several workers (Wade, 1908 ; Da Fano, 1912 ; Murphy, 1926 ; Borghi, 1929 ; Calo, 1932 ; Brown and Pearce, 1922 ; Twort and Lasnitzi, 1938). More recent work confirms and extends these earlier reports which did not always distinguish between primary and transplantable tumours. Black and Speer (1955) noted changes in the lymph nodes of mice bearing spontaneous tumours. Baruah (1960) showed that a transplantable carcinoma and a carcinogen induced sarcoma of the rat were accompanied by splenomegaly and increase in size of the regional lymph nodes. Old, Clark, Benacerraf and Goldsmith (1960) demonstrated splenomegaly in mice bearing sarcoma 180 and Ehrlich ascites tumour. They also noted hepatomegaly and splenomegaly in spontaneous mammary carcinoma. Woodruff and Symes (1962*a*, *b*) noted splenomegaly in mice with newly transplanted mammary carcinoma, and made the interesting observation that the degree of splenomegaly diminished with successive tumour transplants. Lymphoreticular reactions to spontaneous tumours are not common and Parsons (1938) recorded atrophy of lymphoid tissue in 15 mice bearing spontaneous mammary carcinomas.

Our observations have shown that the host response is markedly diminished in the later stages of tumour growth when histological examination gives no indication of the earlier host reaction ; this indicates the importance of serial observations in the assessment of the lymphoreticular response. The collapse of the lymphoreticular response coincides with loss of spleen weight and thymic atrophy. At necropsy the thymus has usually completely disappeared. It begins to atrophy at a time when the tumour is small and the animals show no weight loss and appear to be well. The atrophied thymus showed no pathological change apart from loss of thymocytes. Outbred mice have a lower resistance to this tumour and never show such a severe degree of thymic atrophy. Accordingly this thymic depletion may have an immunological basis caused by the release of increasing amounts of antigen from the neoplasm. Thymic atrophy has been described in tumour bearing rodents by Larionow (1932). Savard and Homberger (1949) showed that thymic atrophy in mice bearing sarcoma 180 was not prevented by hypophysectomy and they concluded that thymic atrophy was not mediated through the pituitary-adrenal axis. Hilf, Burnett and Borrman (1960) showed that the thymic atrophy occurring in Swiss mice bearing Sarcoma 180 was associated with hypertrophy of the adrenal. Begg (1951) made a study of the systemic effects of tumour in rats and noted loss of sudanophilia and cholesterol from the adrenal and loss of weight of the thymus. In our experiments mice bearing the Landschütz tumour showed no definite changes of adrenal weight although more sensitive methods might well reveal changes in that organ.

The carbon clearance method for measuring R.E.S. function was applied to experimental tumours by Biozzi, Stiffel, Halpern and Mouton (1958) who found no alteration of phagocytic function with Ehrlich tumour growing in the peritoneal sac. Stern and Duwelius (1958) showed increased phagocytic function in rats bearing subcutaneous Lewis lymphoma. Our own results showed an initial stimulation of R.E.S. function followed by a return to normal which agrees with the findings of Old, Clark, Benacerraf and Goldsmith (1960) in the case of an ascites tumour of the mouse. The increase in the phagocytic coefficient was suppressed by the passive transfer of cellular immunity in the form of spleen cells, and the likely explanation is suppression of the host response as a result of adoptive immunity. It seems likely to us that the extent of the phagocytic response is largely influenced by the intensity of the host versus graft reaction, especially since Howard (1963) has demonstrated activation of the phagocytic system during a graft versus host reaction.

The foci of immature cells found in the liver probably arise from the endothelium of the hepatic veins and sinusoids. Certainly one can see elongated basophilic cells closely applied to the sinusoidal wall and trace transitions between these cells and the immature mononuclear cells. Mathé *et al.* (1963) have described a large mononuclear cell which arises during the rejection of allogeneic grafts.

The cells of the hepatic parenchyma show an increased number of mitotic figures and binucleate forms. This finding cannot be explained and indicates that the systemic effects of the ascites tumour extends far beyond the lymphoreticular reactions described here.

A constant finding in the spleen has been an increase in size of the Malpighian body and conversion of the small lymphocyte to a larger cell with an open nucleus, nucleoli and basophilic cytoplasm. At this stage of the reaction plasma cells are not present in the spleen although they are found early and in abundance at the periphery of subcutaneous tumour. The splenic changes are identical with those following the injection of bacterial lipopolysaccharide (Stuart and Cooper 1962) and are consistent with antigenic stimulation. The nature and significance of the small pyronophilic spherules in the splenic sinusoids is uncertain. Their morphology and tinctorial properties are similar to those of the cytoplasm of the near-by pyronophilic cells. It is probable that they are fragments of cytoplasm derived from such cells although the possibility that they are an artefact produced during fixation and processing cannot be excluded. If we accept that they are derived from antibody forming cells then the spherules may represent a form of antibody transport.

Polymorphonuclear leucocytosis has long been noted with transplantable tumours (Lewis, 1937 ; Blumenthal, 1941) and developed around the tenth day in our experiments. Although we have failed to isolate aerobic or anaerobic bacteria from our strain of Landschütz tumour and filtrates of ascitic fluid have not produced splenomegaly, bacterial or viral contamination remains a serious hazard in the interpretation of the lymphoreticular reaction to transplanted tumour. Because the activated lymphoreticular cells used in these experiments were able to inhibit or destroy the ascites cells, it seems reasonable to conclude that the histological response described here is directly related to the antigenicity of the tumour.

### SUMMARY

The histology of the lymphoreticular response of strain A mice to the Landschütz ascites tumour has been described and correlated with the R.E.S. phagocytic function as judged by the carbon clearance test. Mitoses in the liver parenchyma and pyronophilic globules in the splenic sinusoids have been described. The initial phase of R.E.S. stimulation is associated with splenomegaly and this is followed by decrease of spleen size and progressive atrophy of the thymus. Treatment with isogeneic immunised lymphoid cells inhibits the phase of R.E.S. stimulation.

We are indebted to Professor G. L. Montgomery for his encouragement and help.

A. M. El Hassan is in receipt of a Scholarship from the University of Khartoum.

A. E. Stuart gratefully acknowledges a research fellowship from the Scottish Hospital Endowment Trust and a generous grant from the British Empire Cancer Campaign for Research.

We wish to thank Mr. J. Paul of the Photomicrography Department for the microphotographs.

### REFERENCES

Baruah, B. D.—(1960) *Cancer Res.*, **20**, 1184.

Begg, R. W.—(1951) *Ibid.*, **11**, 341.

Biozzi, G., Stiffel, C., Halpern, B. M. and Mouton, D.—(1958) *Ann. Inst. Pasteur*, **94**, 681.

Black, M. M. I. and Speer, F. D.—(1955) *Arch. Path.*, **59**, 254.

Blumenthal, H. T.—(1941) *Cancer Res.*, **1**, 196.

Borghi, B.—(1929) *Tumori*, **15**, 289.

Brown, W. H. and Pearce, L.—(1922) *Proc. Soc. exp. Biol., N.Y.*, **20**, 472.

Calo, A.—(1932) *Z. Krebforsch.*, **37**, 151.

Da Fano, C.—(1912) *Sci. Rep. Imp. Cancer Res. Fd*, **5**, 57.

Hilf, R., Burnett, F. F. and Borrman, A.—(1960) *Cancer Res.*, **20**, 1389.

Howard, J. G.—(1963) *Colloq. int. Cent. nat. Rech. sci.*, No. 115, p. 369.

Larionow, L. Th.—(1932) *Z. Krebforsch.*, **37**, 523.

Lewis, M. R.—(1937) *Amer. J. Cancer*, **29**, 510.

Murphy, J. B.—(1926) *Monogr. Rockefeller Inst. Med. Res.*, No. 21.

Mathé, G., Binet, J. L., Seman, G. and Amiel, J. L.—(1963) *Colloq. int. Cent. nat. Rech. sci.* , No. 115, p. 357.

Old, L. J., Clarke, D. A., Benacerraf, B. and Goldsmith, M.—(1960) *Ann. N.Y. Acad. Sc.*, **88**, 264.

Parsons, L. D.—(1938) *J. Path. Bact.*, **47**, 501.

Savard, K. and Homburger, F.—(1949) *Proc. Soc. exp. Biol., N.Y.*, **70**, 68.

Stern, K. and Duwelius, A.—(1958) *Proc. Amer. Ass. Cancer Res.*, **2**, 348.

Stuart, A. E. and Cooper, G. N.—(1962) *J. Path. Bact.*, **83**, 245.

Twort, J. M. and Lasnitzki, M.—(1938) *Endocrinology*, **23**, 87.

Wade, H.—(1908) *J. Path. Bact.*, **12**, 384.

Woodruff, M. F. A. and Symes, M. O.—(1962*a*) *Brit. J. Cancer*, **16**, 707.—(1962*b*) *Ibid.*, **16**, 120.


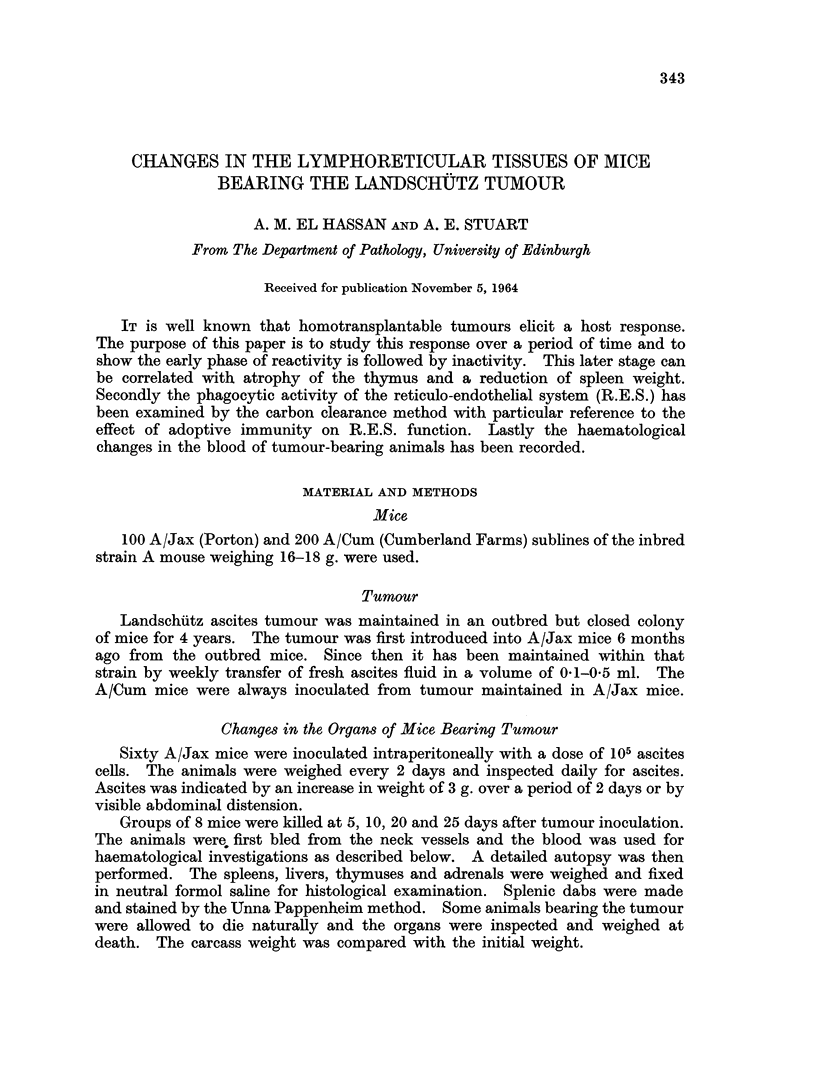

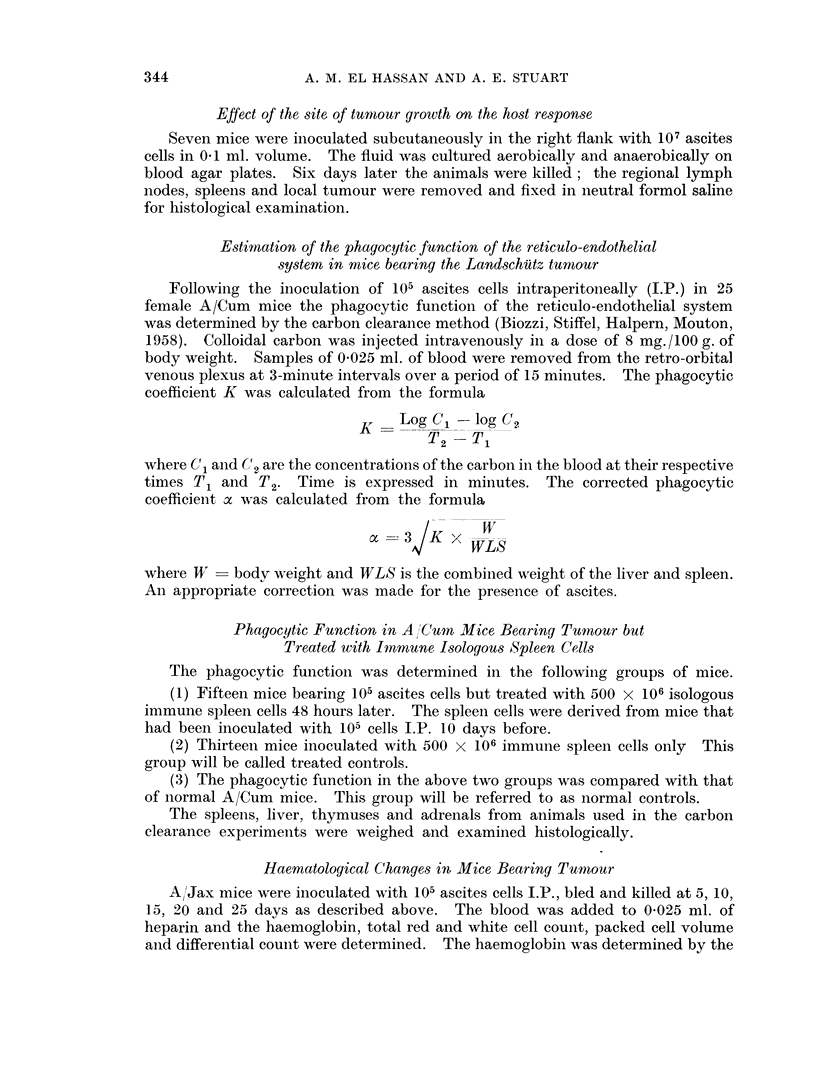

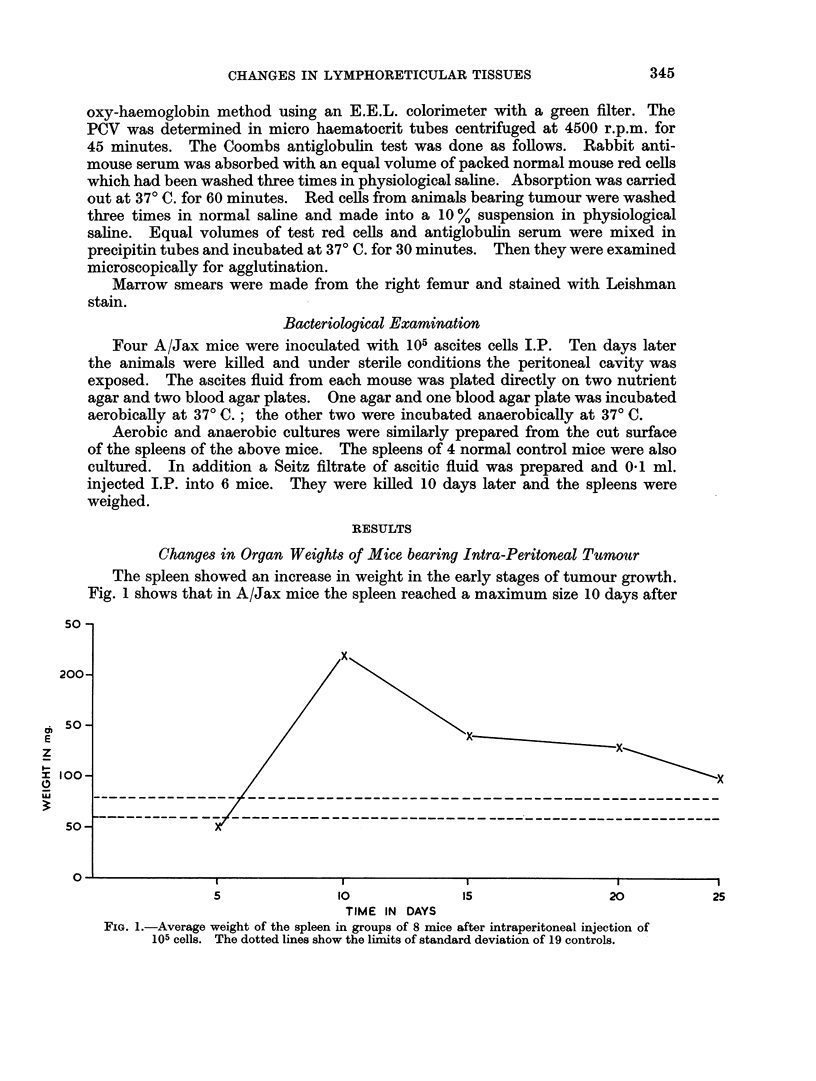

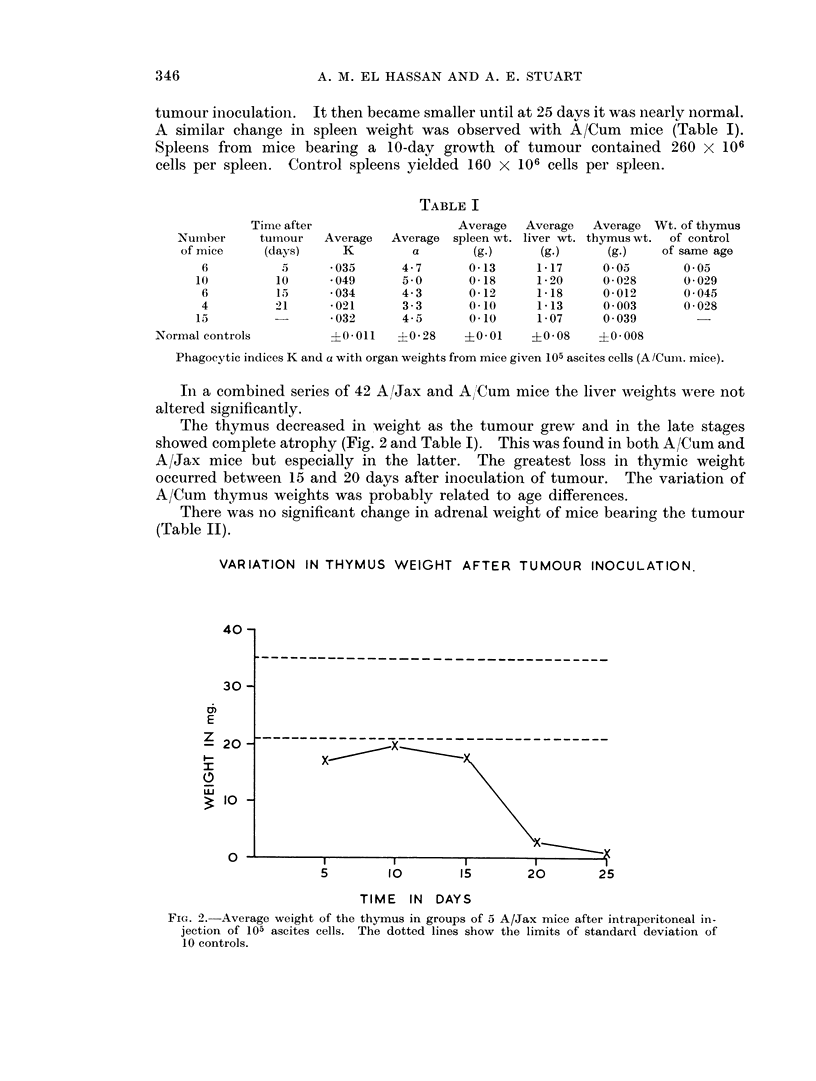

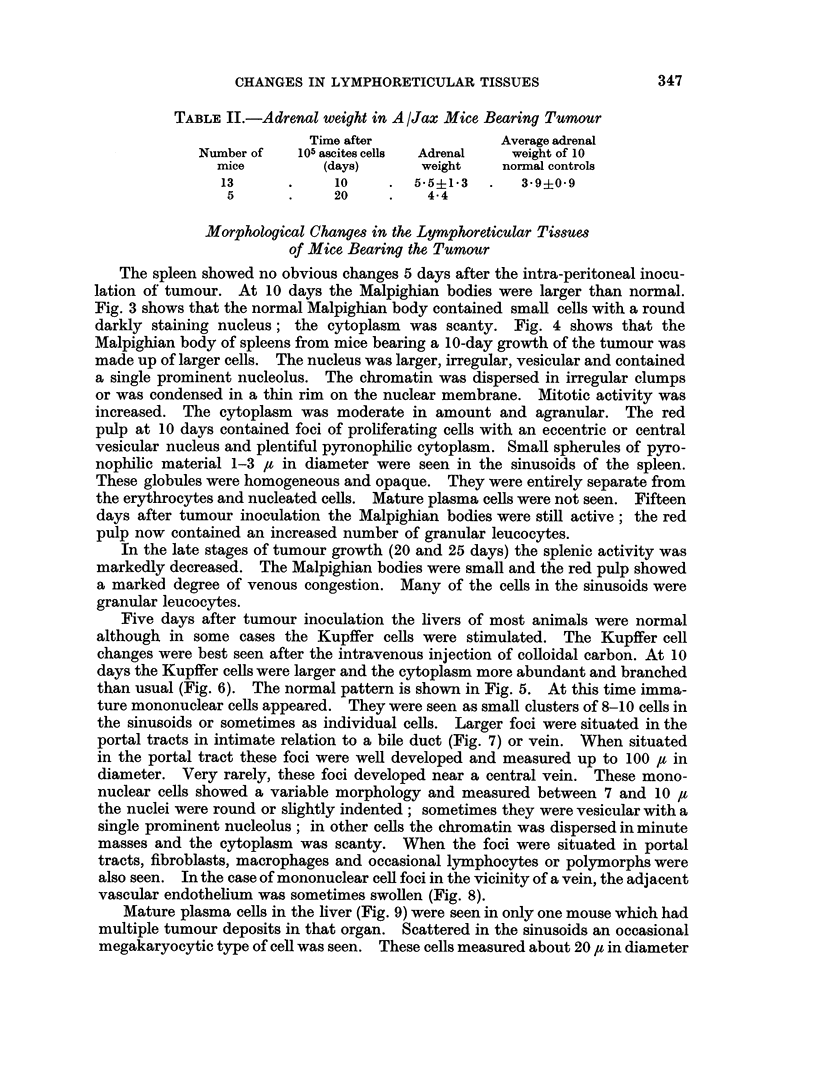

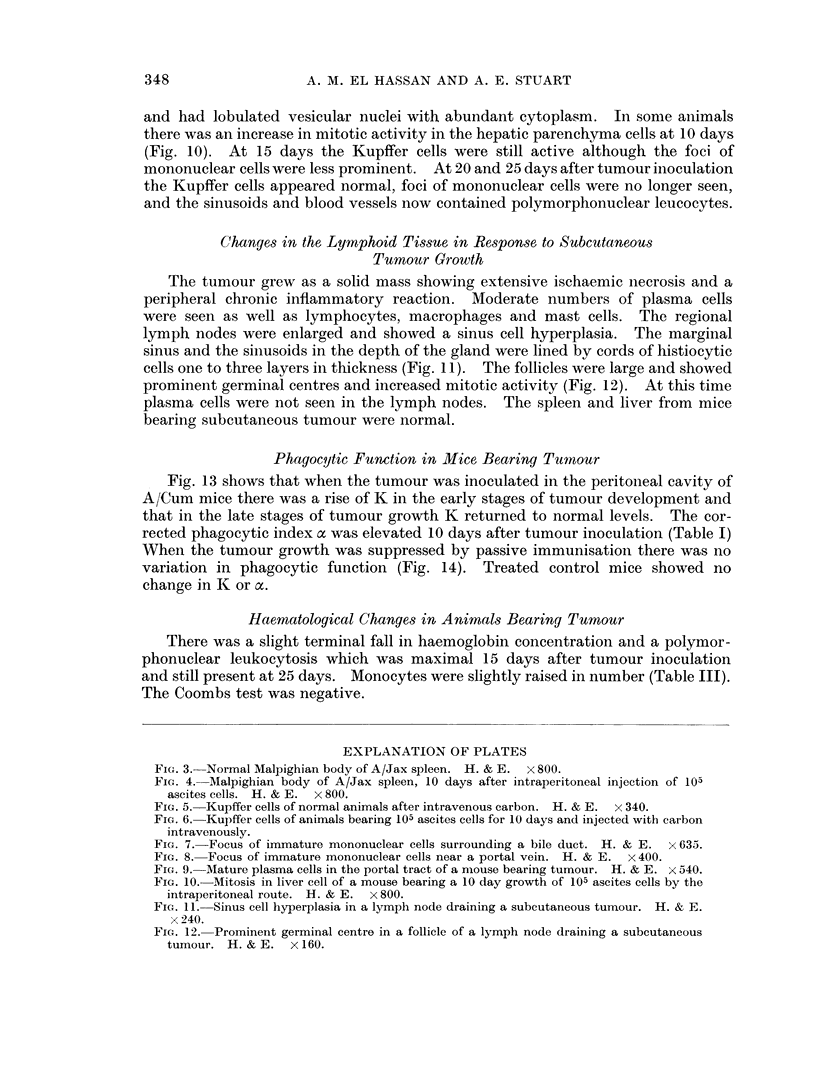

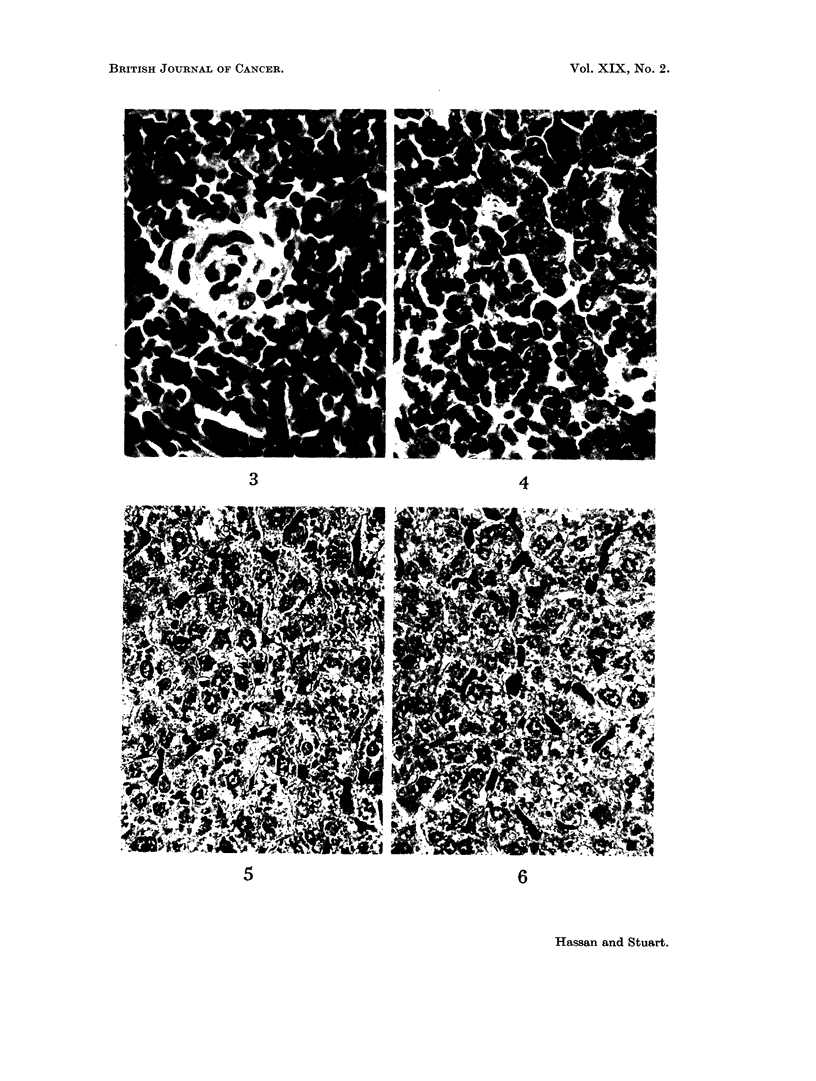

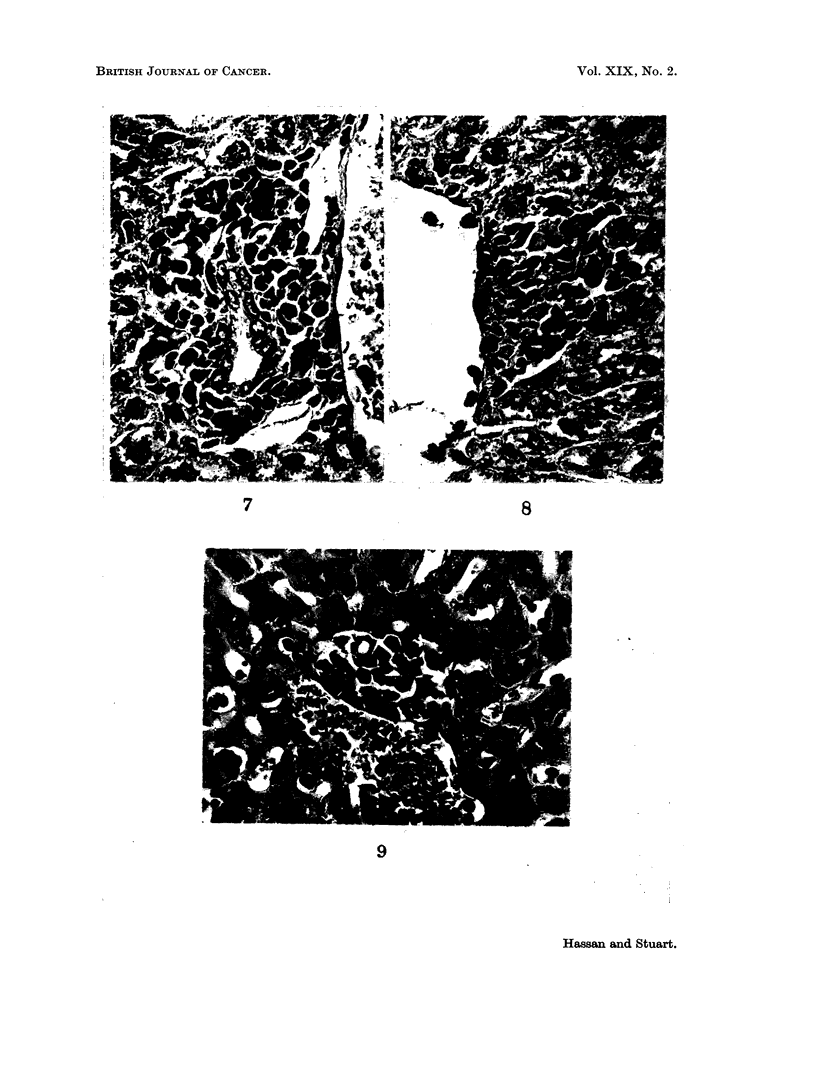

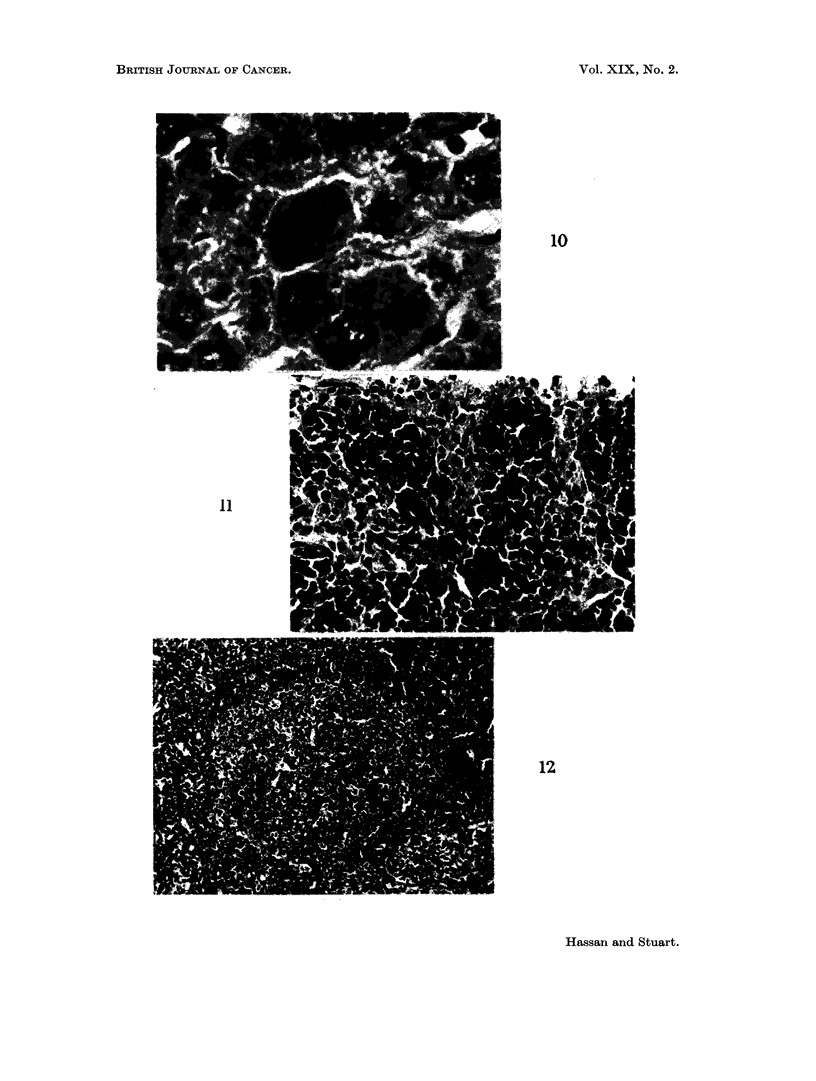

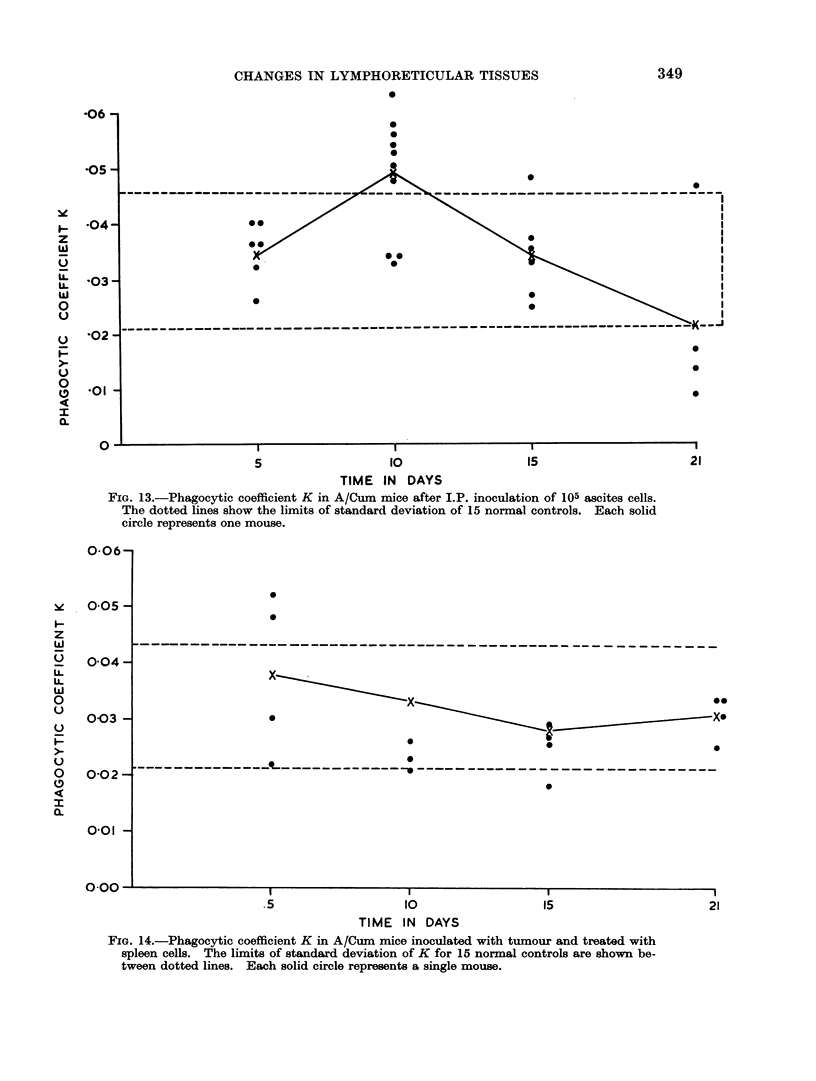

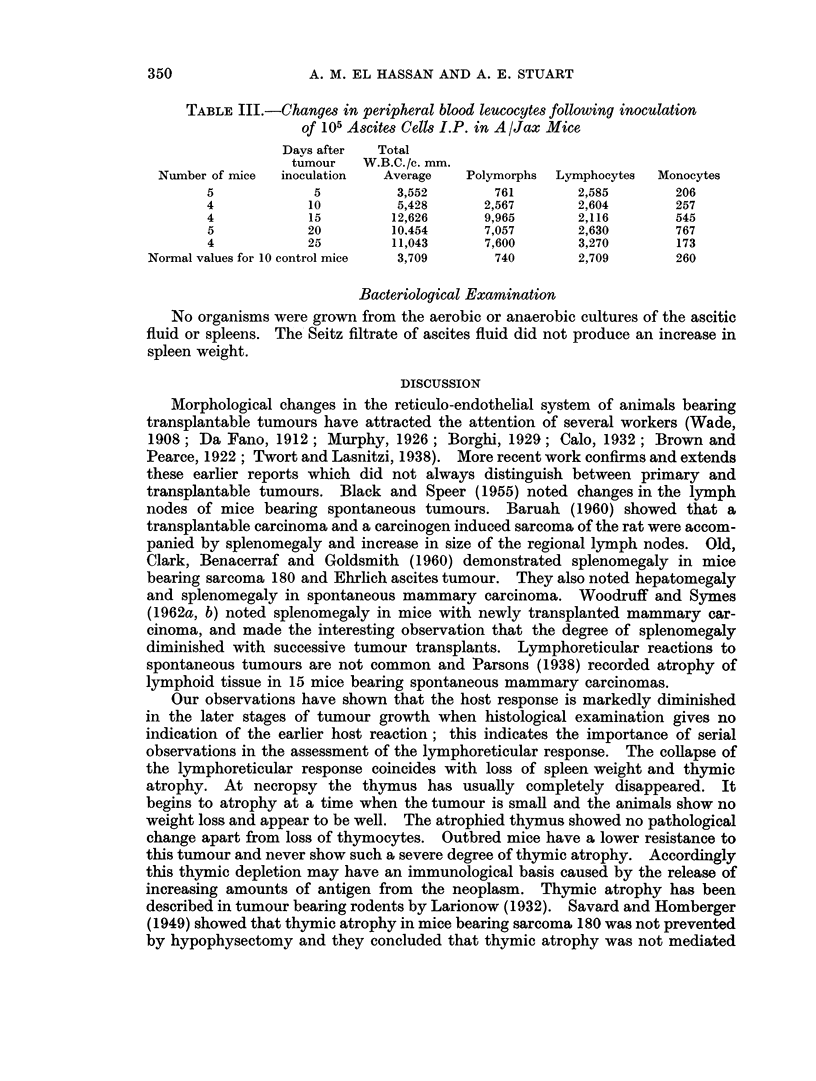

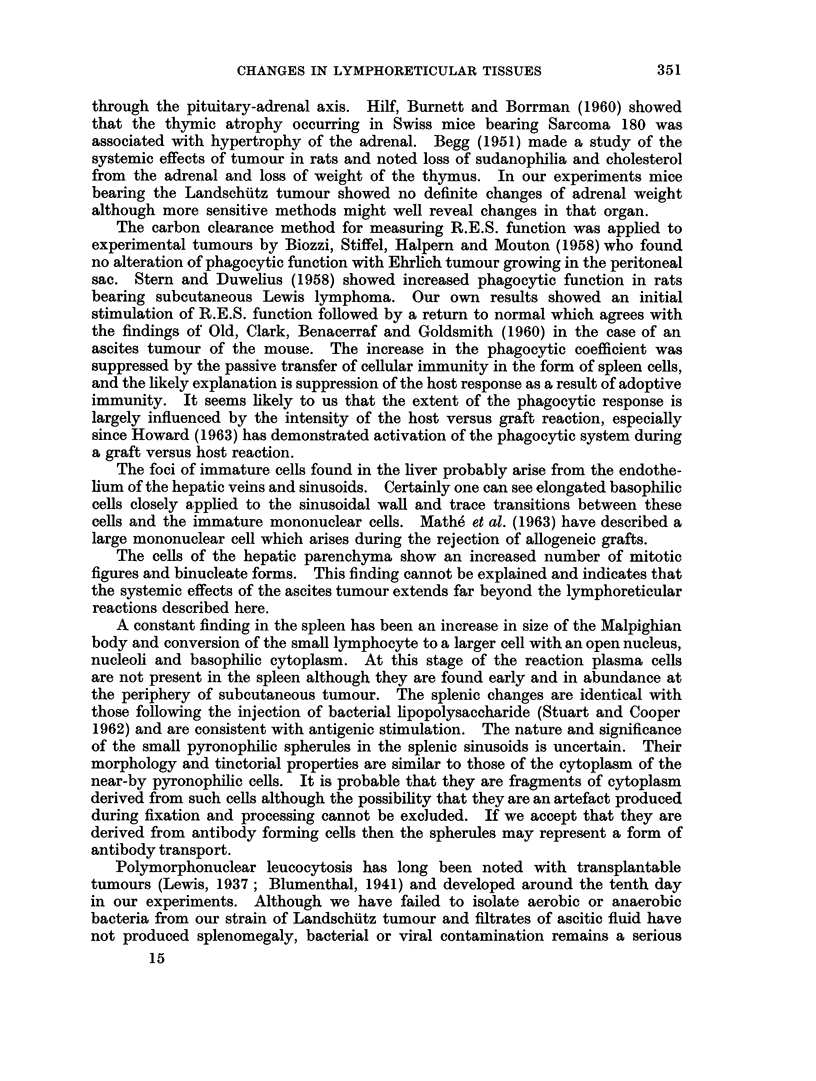

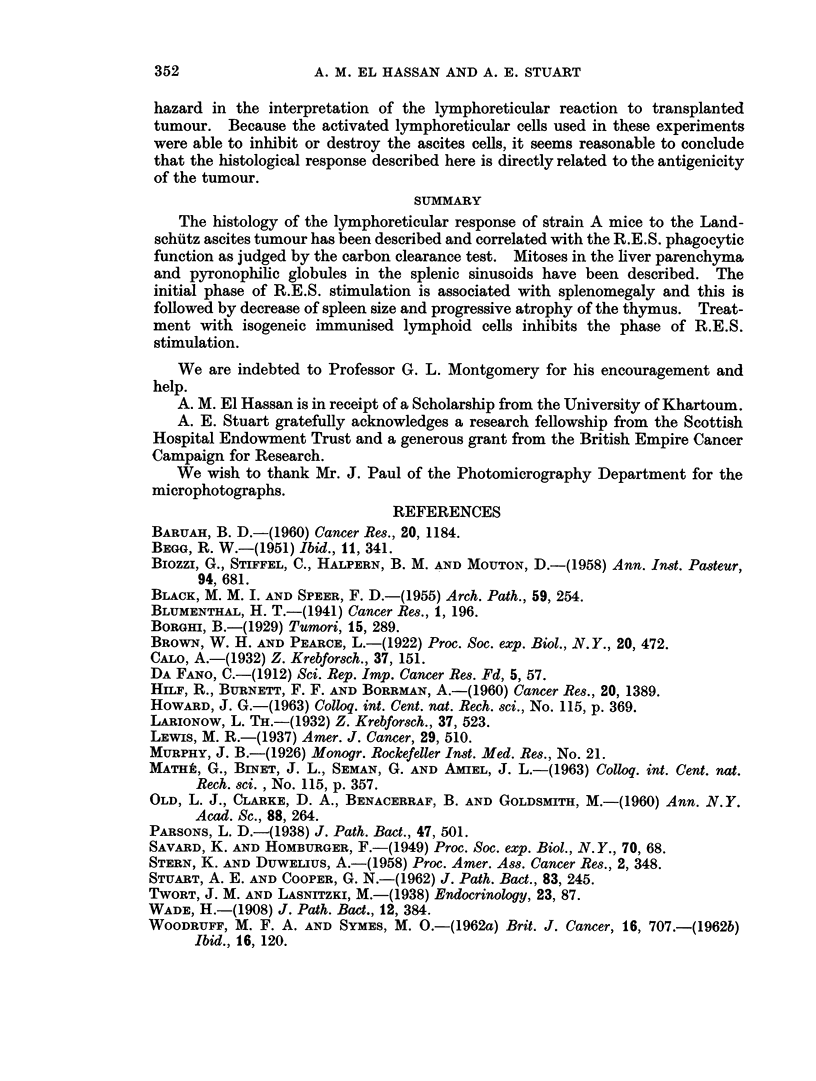


## References

[OCR_00709] BIOZZI G., STIFFEL C., HALPERN B. N., MOUTON D. (1958). Etude de la fonction phagocytaire du S.R.E. au cours du développment de tumeurs malignes expérimentales chez le rat et la souris.. Ann Inst Pasteur (Paris).

[OCR_00711] BLACK M. M., SPEER F. D. (1955). Lymph node structure in control and in tumor-bearing CFW mice.. AMA Arch Pathol.

[OCR_00721] HILF R., BURNETT F. F., BORMAN A. (1960). The effect of sarcoma 180 and other stressing agents upon adrenal and plasma corticosterone in mice.. Cancer Res.

[OCR_00739] STUART A. E., COOPER G. N. (1962). Susceptibility of mice to bacterial endotoxin after modification of reticulo-endothelial function by simple lipids.. J Pathol Bacteriol.

[OCR_00743] WOODRUFF M. F., SYMES M. O. (1962). The use of immunologically competent cells in the treatment of cancer: experiments with a transplantable mouse tumour.. Br J Cancer.

